# Low-Temperature Dielectric Response of Strontium Titanate Thin Films Manipulated by Zn Doping

**DOI:** 10.3390/ma15030859

**Published:** 2022-01-23

**Authors:** Olena Okhay, Paula M. Vilarinho, Alexander Tkach

**Affiliations:** 1 TEMA-Centre for Mechanical Technology and Automation, Department of Mechanical Engineering, University of Aveiro, 3810-193 Aveiro, Portugal; 2 Department of Materials and Ceramic Engineering, CICECO—Aveiro Institute of Materials, University of Aveiro, 3810-193 Aveiro, Portugal

**Keywords:** perovskites, polar dielectrics, thin films, doping, dielectric tunability

## Abstract

The voltage dependence of the dielectric permittivity *ε*′ and the low dielectric loss tan*δ* of incipient ferroelectrics have drawn vast attention to the use of these materials for the development of tuning elements in electronics and telecommunications. Here, we study the DC electric field dependence of low-temperature *ε*′ in ~320 nm thick sol-gel-derived SrTi_1−x_Zn_x_O_3−δ_ thin films with x = 0.01 and 0.05, deposited on Pt/TiO_2_/SiO_2_/Si substrates. Incorporation of Zn onto Ti sites is found to decrease *ε*′ compared to undoped SrTiO_3_ films, while increasing the relative tunability *n_r_* up to ~32.9% under a DC electric field of 125 kV/cm at low temperatures. The hysteresis-free variation in *ε*′ with electric field and tan*δ* values below 0.6% observed for SrTi_1−x_Zn_x_O_3−δ_ film with x = 0.01 make this compound more attractive for tunable device applications.

## 1. Introduction

Dielectric thin films are widely used in modern technology and engineering with the aim of decreasing the size and weight of electronic or communications devices, although the physical phenomena in films are much more complicated to study and to understand than the behaviour of bulk materials. In particular, ferroelectric-related films with DC electric field dependent dielectric permittivity are used to develop frequency-agile electronic devices such as varactors, microwave filters, resonators, voltage-controlled oscillators, and phase shifters, besides passive components [1,2,3,4,5,6,7,8,9,10,11,12,13,14]. For these applications, in addition to the high tunability of dielectric permittivity, the films should have low loss and reduced permittivity values [15,16,17]. Since the tunability of a ferroelectric material is maximized near its Curie point, incipient ferroelectrics such as SrTiO_3_ (ST) are useful at cryogenic temperatures (*T*), whereas regular ferroelectrics such as BaTiO_3_ are more suitable above room *T* [2,15]. Solid solutions of these materials are mainly used for near-room-*T* applications [4,5,9,10,11,12,13,16,18].

The fact that bulk ferroelectrics have the irrevocable disadvantage of needing a very high bias voltage, up to tens of kilovolts, to tune their capacitance results in fully assumed difficulties for applications until the emergence of new materials and film technology. However, because the dielectric loss tan*δ* of thin films is usually considerably higher than that of single crystals (~10^−3^ at 10 GHz for ST [2]) due to point defects, local polar regions, stresses, etc. [15], and increases with the decrease in film thickness [19], applications of films in tunable microwave devices may also be limited. If loss reduction and tunability enhancement in ST-based thin films are to be achieved, devices utilizing films would have a much more attractive performance coupled with the ability to scale down their size. For impedance matching and shorter capacitance-induced delays, a reduced permittivity is also required [17].

The tan*δ* of ST-based materials can be reduced by admixing with low-loss, non-tunable materials [16,20] and by acceptor doping on Ti sites [21,22,23]. Nevertheless, reports of the dielectric response of Zn-doped ST are rather rare and ambiguous. In 2012, Guo et al. reported that the low-*T* dielectric permittivity increases with a Zn content up to 0.9% in Sr_1−x_Zn_x_TiO_3_ ceramics sintered at 1400 °C [24]. In 2020, Pan et al. claimed that the room-*T* permittivity was similar to that of undoped SrTiO_3_ at reduced losses for ceramics with nominal composition SrTi_0.9925_Zn_0.015_O_3_ sintered at 1500 °C [25]. However, the Zn effect in this case is doubtful as Zn is volatile at such high temperatures [26]. Accordingly, the increase in the lattice parameter when Zn was supposed by Pan et al. to be substituted with Nb in their ceramics was observed instead of decrease [25]. The ionic size of Nb^5+^ is smaller than that of Zn^2+^ but larger than that of Ti^4+^ [27]. Therefore, the presence of Zn in the final ceramics should lead to the lattice parameter decreasing with decreasing Zn content, whereas the observed increase can just be explained by Nb substitution for Ti with the absence of Zn in the lattice.

More recently, the room-*T* dielectric permittivity was reported to decrease with increasing ZnTiO_3_ content in sol-gel-derived SrTiO_3_/ZnTiO_3_ heterostructures annealed at just 750 °C by Li et al. [28], thus avoiding the problem of high-*T* Zn volatility. However, no low-*T* dielectric characterization has been reported so far on Zn-doped ST films with Zn incorporation into Ti positions. Therefore, in this work, we performed a compositional, structural, as well as variable temperature and DC electric field dielectric characterisation of sol-gel-derived SrTi_1−x_Zn_x_O_3−δ_ thin films with x = 0.01 and 0.05, deposited on Pt/TiO_2_/SiO_2_/Si substrates, in comparison to similarly prepared films of undoped SrTiO_3_.

## 2. Materials and Methods

For the deposition of SrTi_1−x_Zn_x_O_3__−__δ_ thin films with x = 0, 0.01 and 0.05, solutions with a concentration of about 0.2 M were prepared using strontium acetate C_4_H_6_O_4_Sr (98%, abcr GmbH, Karlsruhe, Germany), tetra-n-butyl orthotitanate C_16_H_36_O_4_Ti (98%, Merck KGaA, Darmstadt, Germany) and zinc acetate-2-hydrate C_4_H_6_O_4_Zn·2H_2_O (99.5%, Riedel-de Haën, Seelze, Germany) as starting precursors. Acetic acid C_2_H_4_O_2_ (99.8%, Merck KGaA, Darmstadt, Germany), 1,2-propanediol C_3_H_8_O_2_ (99.5%, Riedel-de Haën, Seelze, Germany) and absolute ethanol C_2_H_6_O (99.8%, Merck KGaA, Darmstadt, Germany) were used as solvents. Strontium acetate was initially dissolved into heated acetic acid (*T* ~ 60 °C) followed by the addition of zinc acetate-2-hydrate under constant stirring to form a transparent solution. After cooling to room temperature, the former solution was diluted with 1,2-propanediol and then titanium isopropoxide was added. The resultant solution was continuously stirred in a closed flask for 12 h, at the end of which an ethanol was added as a final step. Using these transparent and homogeneous solutions, layers of Zn-doped SrTiO_3_ were deposited on Pt/TiO_2_/SiO_2_/Si substrates (Inostek INC, Seoul, Korea) by spin-coating at 4000 rpm for 30 s, using a spin-coater KW-4A (Chemat Technology, Los Angeles, CA, USA). Before the deposition, the substrates were cleaned in boiling ethanol and dried on a hot plate. After the deposition of each wet layer on the substrate, they were heated on a hot plate at 350 °C for ~1 min to ensure complete removal of the volatile species between the layers. After the complete deposition of the required number of layers (10 layers), they were annealed in air at 750 °C for 60 min with a heating/cooling rate of 5 °C/min.

The thickness of the thin films was determined, and their cross-sectional morphology was observed using scanning electron microscopy (SEM, Hitachi S4100, Tokyo, Japan) under an acceleration voltage of 25 kV. Compositional analysis of the films was performed using an energy dispersive spectroscopy (EDS) system (QUANTAX 75/80, Bruker, Ettlingen, Germany) built in SEM (Hitachi TM4000Plus, Tokyo, Japan) in the top-view geometry under an acceleration voltage of 10 kV. The thin-film crystal phase was analysed at room *T* using a Rigaku D/Max-B X-ray diffractometer (Rigaku, Tokyo, Japan). The X-ray diffraction (XRD) data were recorded in 0.02° step mode with a scanning rate of 1°/min from 20° to 80° using Cu Kα radiation. The lattice parameter was calculated by a least-squares approach Rietveld refinement fitting of the XRD data in the range of 45°–60°. Dielectric spectroscopy and tunability measurements of Zn-doped ST films were performed using Au, sputtered through a mask onto the films, as top electrodes, and the substrate Pt layer as the bottom one. Complex dielectric permittivity, consisting of a real part *ε*′ and an imaginary part *ε*″, as well as the dissipation factor tan*δ* = *ε*″/*ε*′ were measured under an oscillation voltage of 50 mV (and a DC voltage up to 5 V for tunability measurements) at a frequency of 10 kHz, using a precision LCR meter (HP 4284A, Hewlett Packard, Palo Alto, CA, USA). A He closed-cycle cryogenic system (Displex APD-Cryostat HC-2, Allentown, PA, USA) equipped with silicon diode temperature sensors and a digital temperature controller (Scientific Instruments Model 9650, West Palm Beach, FL, USA) was used for *T* variation in the range of 10–300 K.

## 3. Results and Discussion

**Figure 1:**
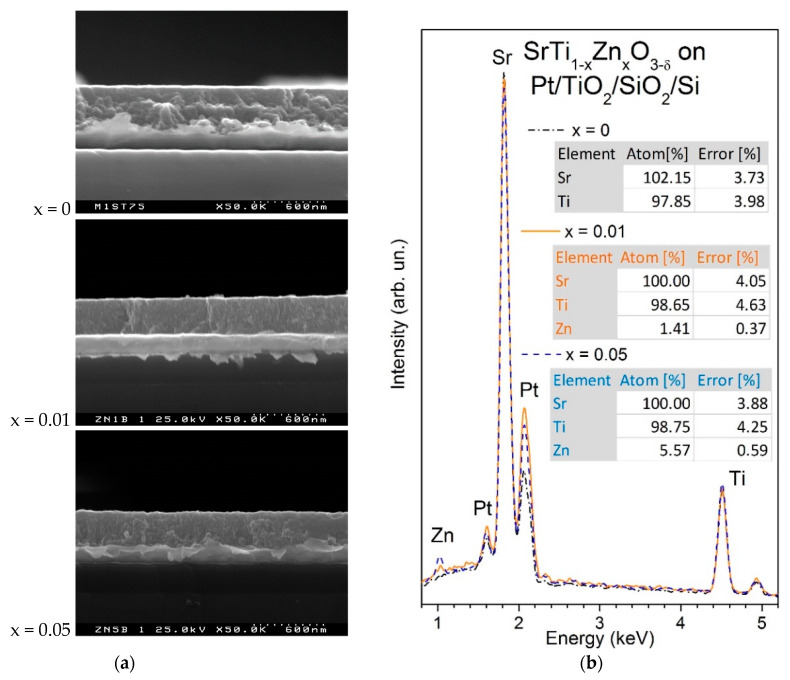
SEM cross-section micrographs (**a**) and energy-dispersive spectra (**b**) for SrTi_1−x_Zn_x_O_3−δ_ thin films with x = 0, 0.01 and 0.05, deposited on Pt/TiO_2_/SiO_2_/Si substrates. The spectra quantification results are also presented in (**b**).

Figure 1a shows the SEM cross-sectional microstructure of SrTi_1−x_Zn_x_O_3__−__δ_ thin films with x = 0, 0.01 and 0.05 grown on platinized silicon substrates. The average film thickness is about 320 nm for both Zn-doped ST films, while that of undoped ST is about 350 nm. Moreover, the morphology of each film reveals several rounded and closely-packed grains across the film’s thickness. EDS analysis of SrTi_1__−__x_Zn_x_O_3__−__δ_ thin films, presented in Figure 1b, clearly displays a Zn peak, whose intensity increases with the value of x. According to the spectra quantification, Zn concentrations in both Zn-doped ST thin films are close to targeted ones, while overall estimated elemental contents indicate the proximity of all the film compositions to nominal ones within error bars.

**Figure 2:**
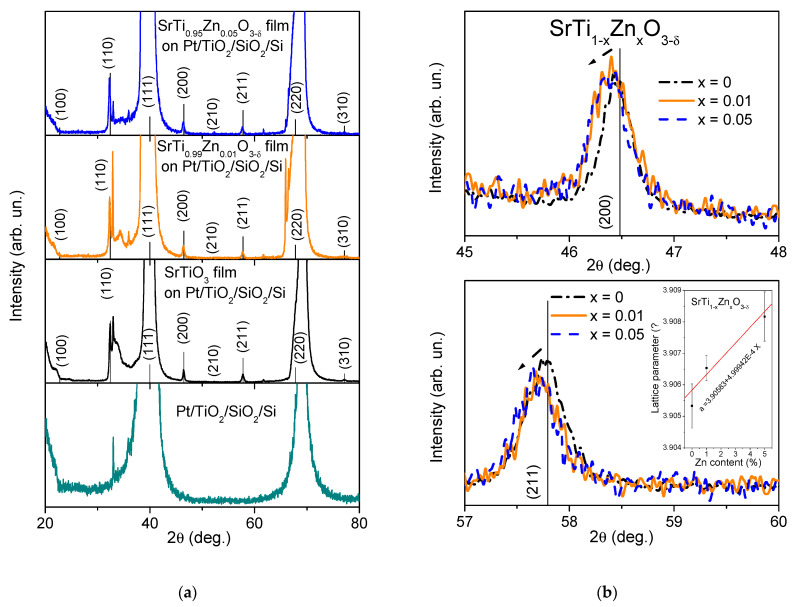
XRD profiles (**a**) and magnified view at reflections (200) and (211) (**b**) for SrTi_1−x_Zn_x_O_3−δ_ thin films with x = 0, 0.01 and 0.05, deposited on Pt/TiO_2_/SiO_2_/Si substrates with reflections related to perovskite structure of SrTiO_3_ (card PDF#35-0734) marked by corresponding indexes. The X-ray diffraction profile for bare Pt/TiO_2_/SiO_2_/Si substrate is also presented in the bottom panel of (**a**). Inset in (**b**) shows the lattice parameter variation with Zn content.

XRD profiles of 1% and 5% Zn-doped and undoped ST films on Pt/TiO_2_/SiO_2_/Si substrates are shown in Figure 2a, revealing only peaks related to the cubic perovskite Pm-3m structure (PDF#35-0734) and those from the substrate layers, particularly Pt, whose XRD profile is also shown in Figure 2a. The cubic perovskite structure peak positions, however, slightly shift toward lower 2θ values with increasing Zn content, as shown in Figure 2b for (200) and (211) peaks, best seen between the Pt peaks. Such a shift implies a lattice parameter increase, as displayed in the inset in Figure 2b. This finding, together with EDS confirmation of the presence of and increase in Zn peaks with nominal Zn concentration and an educated guess of similar strain in the films [29], proves the incorporation of Zn onto the Ti sites of the SrTiO_3_ lattice, at least in its major part. The proof can be found by taking into account that the ionic size of Sr^2+^ is larger than that of Zn^2+^, whereas the ionic size of Zn^2+^ is larger than that of Ti^4+^ [27]. Therefore, it is reasonable to suppose the formation of a SrTi_1__−__x_Zn_x_O_3__−__δ_ solid solution, since, for Sr_1__−__x_Zn_x_TiO_3_ solid solutions, the lattice parameter should decrease with Zn content. Moreover, the lattice parameter variation slope is similar to that reported for SrTi_1__−__x_Mg_x_O_3__−__δ_ ceramics [30]. Such similarity is also reasonable, since Mg^2+^ possesses an ionic size close to that of Zn^2+^ [27].

**Figure 3:**
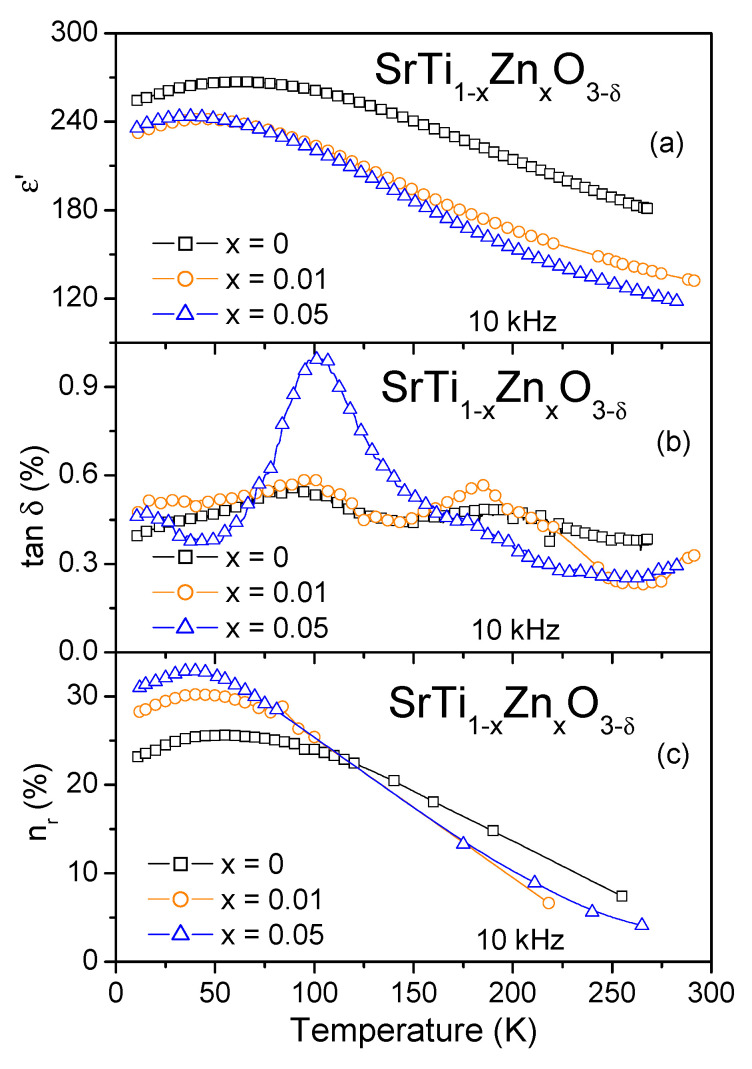
Temperature dependence of the real part of dielectric permittivity *ε*′ (**a**), dissipation factor tan*δ* (**b**), and relative tunability *n_r_* (**c**) under a 125 kV/cm *dc* electric field at 10 kHz for SrTi_1−x_Zn_x_O_3−δ_ thin films with x = 0.01 and 0.05 in comparison to that for SrTiO_3_ film.

The *ε*′(*T*) dependence of the reference ST thin film shown in Figure 3a reveals an increase in cooling until a diffuse peak at about 60 K. This peak is also seen in the *ε*′(*T*) of SrTi_1__−__x_Zn_x_O_3__−__δ_ films with peak temperatures decreasing to about 46 K for x = 0.01 and 38 K for x = 0.05. The permittivity value also decreases with increasing Zn content. The permittivity decrease was also observed for SrTi_1__−__x_Mg_x_O_3__−__δ_ ceramics [30] and thin films [31] as well as for SrTi_1__−__x_Mn_x_O_3_ ceramics [32], being explained by the substitution of smaller and highly polarizable Ti^4+^ by larger and hence less polarizable dopant ions. Such an explanation should also be valid in the case of Zn doping on Ti sites of ST. The temperature dependence of the dissipation factor of Zn-doped ST films shown in Figure 3b presents up to three peaks at ~17, ~100, and ~185 K. The tan*δ* magnitude is rather low, staying between 0.3% and 0.6% for all the films, with the only exception being a tan*δ* value increase up to 1% at around 100 K for SrTi_0.95_Zn_0.05_O_3__−__δ_ film.

On the other hand, reduced permittivity is desirable for impedance matching of tunable dielectric components as well as for a decrease in capacitance-induced delays [17]. Therefore, we checked the dielectric tunability of Zn-doped ST films under 125 kV/cm at 10 kHz and compared it to that of similarly prepared undoped SrTiO_3_ film, as shown in Figure 3c. For that, the relative tunability was calculated by the equation:

*n_r_* = [*ε*′(0) − *ε*′(*E*)]/*ε*′(0) × 100% (1)

where *ε*′(*E*) and *ε*′(0) denote the real part of dielectric permittivity under the applied bias field *E* and after returning to zero field, respectively. As seen in Figure 3c, the temperature variation of *n_r_* shows the peak similar to that observed in *ε*′(*T*) dependence with the peak temperature decreasing with increasing Zn content. Concerning the relative tunability values, they are close to each other for all the films under study in the *T* range above 100 K and are evidently enhanced by Zn doping below 100 K. As a result, the peak tunability value grows from ~25.6% at ~55 K for undoped SrTiO_3_ to ~30.2% at ~40 K for SrTi_0.99_Zn_0.01_O_3__−__δ_ and further to ~32.9% at ~40 K for SrTi_0.95_Zn_0.05_O_3__−__δ_ films.

**Figure 4:**
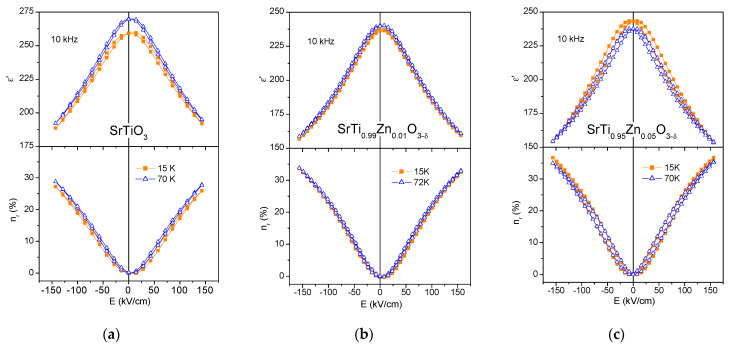
DC electric field dependence of the real part of dielectric permittivity *ε*′ (top panel) and relative tunability *n_r_* (bottom panel) for SrTi_1−x_Zn_x_O_3−δ_ thin films with x = 0 (**a**), 0.01 (**b**) and 0.05 (**c**) at 10 kHz and selected temperatures.

Figure 4 shows *ε*′ and *n_r_* of 1% and 5% Zn-doped and undoped ST films as a function of the applied DC electric field at selected temperatures of about 15 and 70 K. In agreement with Figure 3, at these temperatures, the permittivity is lower and tunability is higher for Zn-doped ST films compared to the undoped one, whose curves are shown in Figure 4a. Moreover, SrTi_0.99_Zn_0.01_O_3__−__δ_ film presents almost hysteresis-free *ε*′(*E*) variation (see Figure 4b), while some hysteresis is observable for SrTi_0.95_Zn_0.05_O_3__−__δ_ film (see Figure 4c). Since the hysteresis can make tunable device operation ambiguous, 1% Zn-doped ST film is more recommended for tunable component applications.

## 4. Conclusions

Zn doping was successfully performed in ~320 nm thick sol-gel-derived ST films deposited on Pt/TiO_2_/SiO_2_/Si substrates, as confirmed by EDS and XRD analysis, and found to have a significant effect on the dielectric response of SrTi_1−x_Zn_x_O_3−δ_ films with x = 0.01 and 0.05. The observation of Zn peaks by EDS and the increase in lattice parameter with Zn content confirmed the substitution of Ti^4+^ by Zn^2+^ ions. Due to such substitution, the relative tunability was found to increase compared to that of undoped ST film at low temperatures, reaching a value of up to 32.9% under a DC electric field of 125 kV/cm. Although the low-*T* tunability was the highest for SrTi_0.95_Zn_0.05_O_3__−__δ_ thin film, the hysteresis-free electric field dependence of the permittivity together with the dissipation factor below 0.6% observed in SrTi_0.99_Zn_0.01_O_3__−__δ_ film make the latter film more relevant for tunable device applications.

## Data Availability

The data presented in this study are available on request from the corresponding authors.
